# The ionotropic AMPA receptor contributes to autoimmunity via altered regulatory T cell differentiation

**DOI:** 10.1016/j.isci.2025.114267

**Published:** 2025-11-27

**Authors:** Marisa Mitchell-Flack, Makenzie Higgins, Ying Zheng, Ada Tam, Hana Goldschmidt, Hiroshi Nishio, Bian Liu, Christopher M. Cherry, Michael Patatanian, Richard Blosser, Sung Soo Mun, Aditya Suru, Richard Huganir, Hong Yu, Drew Pardoll

**Affiliations:** 1 Bloomberg∼Kimmel Institute for Cancer Immunotherapy, Immunology and Hematopoiesis Division, Department of Oncology, Johns Hopkins University School of Medicine, Baltimore, MD, USA; 2 The Sidney Kimmel Comprehensive Cancer Center, Johns Hopkins University School of Medicine, Baltimore, MD, USA; 3 Department of Neuroscience, Kavli Neuroscience Discovery Institute, Johns Hopkins University School of Medicine, Baltimore, MD, USA; 4 CM Cherry Consulting, Baltimore, MD, USA

**Keywords:** Immunology, Immune system, Immune response, Stem cells research

## Abstract

The AMPA receptor (AMPAR) is an ionotropic glutamate receptor that is essential for neuronal communication, yet its role in the immune system remains poorly understood. Here, using a *CD4Cre* selective deletion mouse model, we provide the first functional characterization of AMPAR deficient T cells. We demonstrate that AMPAR deletion in T cells significantly protects against severe paralysis in an experimental autoimmune encephalomyelitis (EAE) model, and this protection is associated with increased regulatory T cell (Treg) presence within the spinal cord. *In vitro* studies reveal that the deletion of the AMPAR intrinsically promotes Treg generation. Mechanistically, AMPAR deletion increases IL2 signaling and activates the mTORC1 pathway, supporting Treg development and function. These novel findings suggest that a function of the AMPAR in CD4 T cells is to limit immune suppression by restricting Treg differentiation. Targeting AMPARs on T cells could offer a novel therapeutic approach for the treatment of autoimmune disease.

## Highlights

• AMPAR deletion in T cells or blockade protects against autoimmune neuroinflammation
• Loss of AMPAR signaling promotes inducible Treg (iTreg) differentiation
• AMPAR deficiency enhanced IL2/STAT5 signaling, glycolysis, and lipid synthesis

## Graphical abstract

**Figure:**
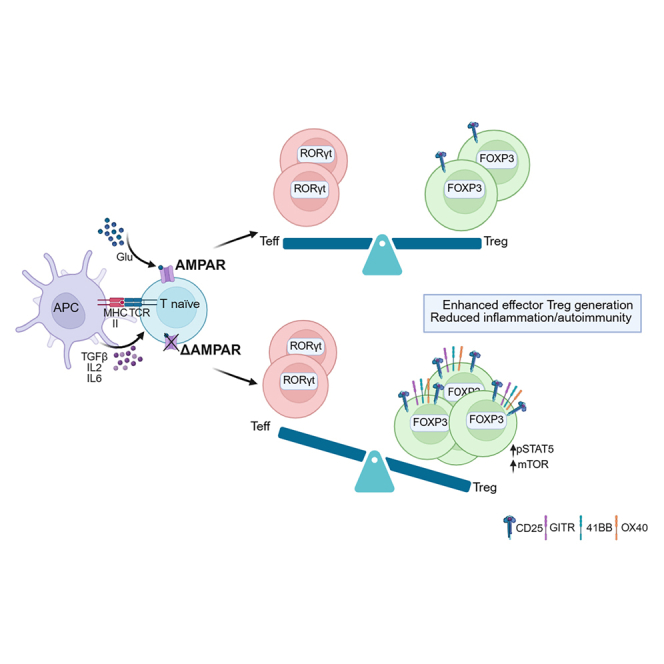

## Introduction

Autoimmunity is defined as the significant breakdown in immune tolerance and consequently the targeted destruction of self-tissue by immune cells. CD4 T cells are known to contribute both pathogenic and protective roles in this context. Effector T cell (Teff) subsets such as Th1 and Th17 produce proinflammatory cytokines which can exacerbate inflammation. In contrast, regulatory T cells (Tregs) employ several mechanisms to suppress autoreactive T cell responses and maintain self-tolerance.^1^ The loss of Treg differentiation and function alone can cause a deleterious breakdown in self-tolerance and result in severe systemic damage and even premature death.^2^ Indeed, for autoimmune diseases such as multiple sclerosis (MS), it is known that this balance is tipped toward the pro-inflammatory Teff state and away from suppressive Treg presence and function.^3^^,^^4^^,^^5^ Therefore, elucidating mechanisms that enhance Treg differentiation and function to counter the disease-induced shift toward Teff functionality is critical to improving immunotherapies for autoimmune disease.

The AMPAR is a heterotetramer comprised of four possible subunits: GluA1, GluA2, GluA3, and GluA4 and is known to be expressed on post-synaptic neurons.^6^ In neurons, the binding of extracellular glutamate to the AMPAR facilitates the opening of the channel pore to permit the ion flux of sodium (Na^+^), potassium (K^+^), and calcium (Ca^2+^) causing a membrane potential change to drive synaptic transmission.^7^ Glutamate is not only an important neurotransmitter in the neuronal system, but also a metabolite from glutamine metabolism actively secreted by proliferating cells in the tissue environment.^8^^,^^9^ In the immune system, previous reports have linked AMPAR activity with inflammation and autoimmunity. Specifically, increased expression of AMPAR subunits on T cells was observed among patients with relapsing multiple sclerosis.^10^^,^^11^ Furthermore, AMPAR antagonists have also shown promise in reducing inflammation in preclinical models of osteoarthritis, viral encephalomyelitis, and MS.^12^^,^^13^^,^^14^ However, within each of these studies, the effect of AMPAR antagonism was determined primarily by reduced clinical signs of inflammation and not by the evaluation of specific immune cell contributions. While these studies show that AMPAR antagonism reduces inflammation, whether this antagonism functions through the alteration of T cell-specific functions of the AMPAR remains unknown. Moreover, what role the AMPAR has in regulating T cell function has not been assessed.

To this end, we sought to investigate the role of the AMPAR in T cells in autoimmunity using a genetic approach. We observed that the T cell specific deletion of the AMPAR protected mice from developing severe autoimmune disease in a preclinical model of MS. This protection was marked by the enhanced presence of effector Tregs in the spinal cords of these mice. Further analysis revealed that AMPAR-deficient T cells exhibit enhanced differentiation into Treg cells based on the increased expression of forkhead box protein 3 (Foxp3) as well as these key regulators: CD25, 4-1BB, OX40, and GITR. In support of increased Treg differentiation, we observed a significant upregulation of the mTORC1 and lipid metabolism pathways among AMPAR-deficient Tregs. These data identify a novel T cell specific role for the AMPAR through the regulation of Treg differentiation and function, and whose targeted inhibition can potentially improve autoimmune disease outcomes.

## Results

### T cell specific deletion of the AMPAR protects mice from severe experimental autoimmune encephalomyelitis

The AMPAR is expressed in T cells (Figure S1A).^15^^,^^16^ To characterize the T cell intrinsic role of the AMPAR, we crossed GluA1^*fl/fl*^GluA2^*fl/fl*^GluA3^*fl/fl*^ triple floxed mice to CD4Cre mice to generate GluA1^*fl/fl*^GluA2^*fl/fl*^GluA3^*fl/fl*^ CD4Cre^+^ (T^ΔAMPAR^) mice.^17^^,^^18^^,^^19^ To confirm the deletion of the AMPAR, we evaluated *Gria2* and *Gria3* mRNA expression in T^ΔAMPAR^ CD4 T cells by qPCR. Expression of these subunits in T^ΔAMPAR^ CD4 T cells was absent (Figure S1B). For the duration of our studies, we utilized GluA1^*fl/fl*^GluA2^*fl/fl*^GluA3^*fl/fl*^CD4Cre^−^ mice as wild-type littermate controls (WT). T^ΔAMPAR^ mice did not exhibit any developmental abnormalities and are similar in physical size to their littermate controls (data not shown). T cell development was unaffected by AMPAR deletion as T^ΔAMPAR^ mice exhibit normal frequencies of both single and double-positive thymocytes as well as normal peripheral frequencies of CD4 and CD8 T cells (Figures S1C–S1E).

**Figure 1:**
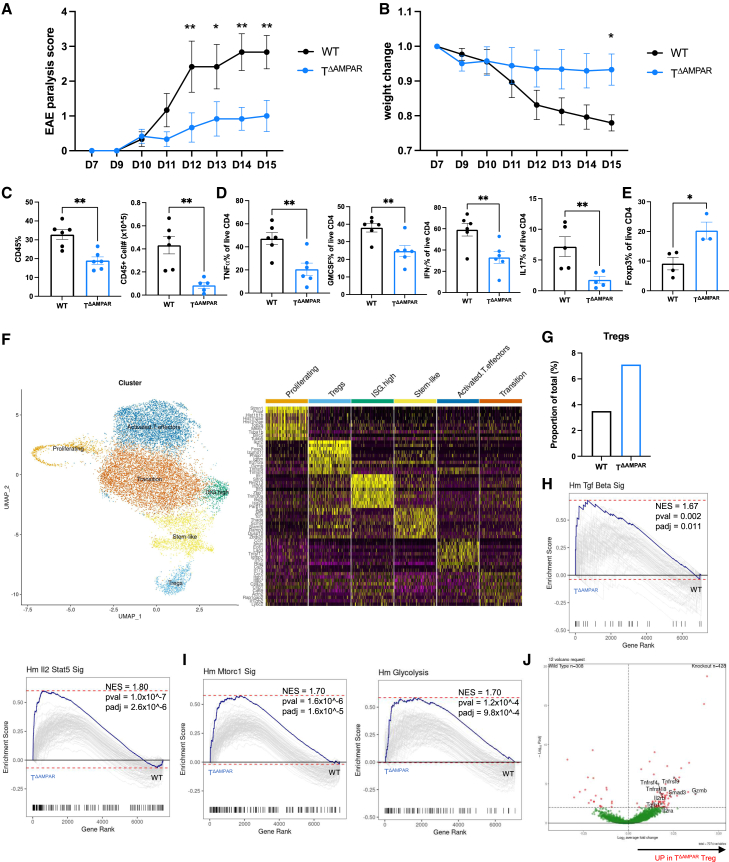
T^ΔAMPAR^ mice are protected from developing severe autoimmune disease, and this is associated with an enhanced effector Treg presence WT and T^ΔAMPAR^ mice were immunized with CFA-MOG and injected with PTX to induce EAE. (A) EAE paralysis scores and (B) weight change over the course of disease of WT and T^ΔAMPAR^ mice (mean ± SEM, ANOVA, representative of more than 3 independent experiments with 4–6 biological replicates per group). (C–E) Spinal cord infiltrating immune cells were isolated and evaluated by flow cytometry on day 15. (C) Quantification of CD45^+^ frequency and total CD45^+^ cell number, (D) quantification of frequency of the CD4^+^ TNF⍺, GMCSF, IFN*γ*, IL17, and (E) Foxp3 (mean ± SEM, ANOVA, representative of more than 3 independent experiments with 4–6 biological replicates per group). (F–I) Live CD4 T cells were isolated and pooled from the spinal cords of WT and T^ΔAMPAR^ mice with EAE at day 14, and scRNAseq was performed. (F) 2-D UMAP projection of WT and T^ΔAMPAR^ CD4 T cells. Unique clusters are color coded and annotated with cluster names (left). Heatmap depicts the relative expression of the top 5 differential genes for each cluster (right). (G) Proportion of WT and T^ΔAMPAR^ CD4 T cells found within the Treg cluster. (H and I) GSEA plots of T^ΔAMPAR^ versus WT cells in the Treg cluster. (H) Hallmark (Hm) Tgf beta signaling (top) and IL2 Stat5 signaling (bottom), (I) Hm mTORC1 signaling (left) and Hm glycolysis (right). (J) Volcano plot highlights effector Treg associated genes upregulated in T^ΔAMPAR^ Treg cluster 3 cells. Statistical significance is represented as ns > 0.05, ∗*p* < 0.05, ∗∗*p* < 0.01.

To study the role of the AMPAR in T cell tolerance, we chose to evaluate whether AMPAR deficiency in T cells would affect outcomes in an *in vivo* experimental autoimmune encephalomyelitis (EAE) disease model. Upon the induction of EAE, we evaluated paralysis progression and observed that T^ΔAMPAR^ mice had significantly reduced EAE scores and weight loss over the course of disease (Figures 1A and 1B). Consistent with their reduced disease state, at the peak of disease, T^ΔAMPAR^ mice had significantly fewer spinal cord infiltrating immune cells (Figure 1C). EAE pathogenesis is associated with the spinal cord infiltration of proinflammatory CD4 T helper (Th) 1 and Th17 cells, particularly those that express high levels of proinflammatory cytokines.^4^ Our assessment of the spinal cord infiltrating T^ΔAMPAR^ CD4 T cells revealed these cells consistently had reduced expression of TNFα, GMCSF, IFNγ, and IL-17 pro-inflammatory cytokines (Figure 1D). In contrast to the pathogenic role of Th1 and Th17 cells in EAE, Tregs have been shown to suppress aberrant inflammation and thereby provide protection from severe EAE.^4^ Given that the infiltrating T^ΔAMPAR^ CD4 T cells had reduced expression of proinflammatory cytokines, we also assessed whether Treg presence was enhanced within T^ΔAMPAR^ spinal cords. Indeed, we noted that CD4^+^Foxp3^+^ cells were higher in frequency within T^ΔAMPAR^ spinal cords (Figure 1E). Despite these observations within the spinal cords of T^ΔAMPAR^ mice, there were no differences observed in inflammatory Th1, Th17, or anti-inflammatory Treg CD4 T cells derived from draining lymph nodes (dLNs) at the peak of disease (Figures S1F and S1G). These data suggested that a deficiency of the AMPAR in T cells is sufficient to protect mice from severe EAE. Furthermore, this protection correlated with the reduced proinflammatory state of spinal cord infiltrating CD4 T cells and the increased presence of Tregs.

To begin to investigate the molecular pathways associated with the protection from severe EAE observed in T^ΔAMPAR^ mice, we isolated and pooled spinal cord infiltrating WT and T^ΔAMPAR^ CD4 T cells at the peak of EAE disease and submitted them for single cell RNA sequencing (scRNAseq). After quality control filtering, the integration of 14,801 high quality CD4 T cells identified six clusters displayed by Uniform manifold approximation and projection (UMAP) and annotated by their unique gene expression (Figure 1F). Analysis of the CD4 distribution among clusters showed a nearly 2-fold higher ratio of T^ΔAMPAR^ CD4 T cells within the Treg cluster compared to WT (Figure 1G). CD4 distribution among T conventional (Tconv) clusters did not reveal a striking difference between WT and T^ΔAMPAR^ CD4 T cells (Figure S1H). The enhanced distribution of T^ΔAMPAR^ in the Treg cluster corroborated the previous observations of enhanced Treg presence via flow cytometry analysis of spinal cord infiltrates (Figure 1E). Moreover, gene set enrichment analysis (GSEA) revealed that the greatest transcriptomic differences between WT and T^ΔAMPAR^ CD4 cells were found within the Treg cluster. GSEA analysis revealed T^ΔAMPAR^ Tregs had enriched expression of genes associated with the hallmark pathways for TGFβ and IL-2 signaling, which are involved in both the differentiation and maintenance of Tregs (Figure 1H).^20^^,^^21^ T^ΔAMPAR^ Treg cells also had upregulated mTORC1 and glycolysis pathway enrichment, which has been previously associated with an effector Treg functional state (Figure 1I).^22^^,^^23^^,^^24^ Further support for an increased effector Treg state was evidenced by the increased expression of *Gzmb* (Granzyme B), *Tnfrsf9* (4-1BB), *IL2ra* (CD25), *Tnfrsf4* (OX40), *Tgfb1* (TGFβ1), and *Tnfrsf18* (GITR) among T^ΔAMPAR^ Tregs (Figure 1J). These molecules have been shown to be associated with the enhanced activation state and suppressive capacity of Tregs.^1^^,^^25^^,^^26^^,^^27^^,^^28^ Taken together, our data suggest that protection from EAE in mice with a T cell specific deletion of the AMPAR is associated with enhanced effector Treg cell responses. Under EAE disease conditions, we observed this significant change in the proportion and transcriptional profile of T^ΔAMPAR^ Treg cells within the spinal cord. Therefore, we focused primarily on understanding the role of the AMPAR in Treg differentiation and function.

### T cell specific deletion of the AMPAR enhances Treg polarization

**Figure 2:**
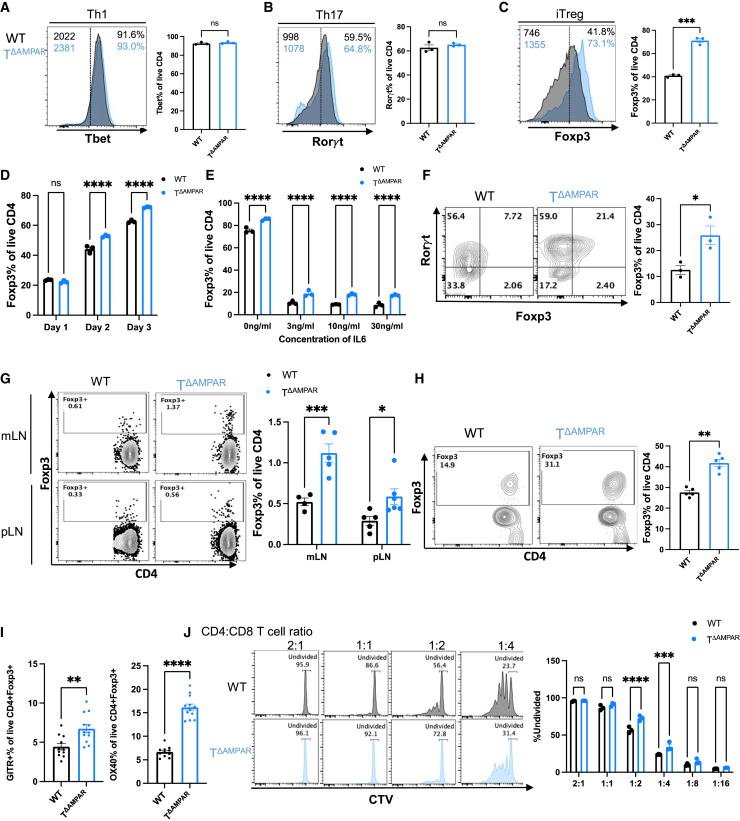
AMPAR deficiency preferentially polarizes CD4 T cells to Tregs (A–D) Naive CD4 T cells were activated with 5 μg/ml anti-CD3 and 2 μg/ml anti-CD28 under Th1, Th17, and iTreg polarizing conditions. Cells were analyzed on day 3. Representative histogram of lineage defining transcription factor expression with gMFI and frequency annotation (left) and frequency quantification (right) for (A) Th1, (B) Th17, and (C) iTreg cells (mean ± SEM, unpaired *t* test, representative of more than 3 independent experiments). (D) Foxp3 expression over the course of iTreg differentiation *in vitro* (mean ± SEM, ANOVA, representative of 2 independent experiments). (E) Naive CD4 T cells were isolated and cultured under iTreg polarizing conditions and a titration of IL6. Quantification of CD4^+^Foxp3^+^ frequency (mean ± SEM, ANOVA, representative of 2 independent experiments). (F) Representative histogram of Foxp3 expression (left) and quantification (right) for Th17 cells (mean ± SEM, unpaired *t* test, representative of more than 3 independent experiments). (G) Naive CD4 T cells were isolated from WT and T^ΔAMPAR^ mice and adoptively transferred to Rag2^−/−^ mice. Representative flow plots of Foxp3^+^CD4^+^ expression (left) and quantification (right) of week 3 harvested mLN (top) and pLN (bottom) (mean ± SEM, ANOVA, representative of 3 independent experiments). (H) WT and T^ΔAMPAR^ mice were immunized with CFA-MOG, and their dLN were isolated and restimulated with 50 μg/ml MOG peptide under iTreg culture conditions. Representative flow plot (left) and quantification (right) of CD4^+^Foxp3^+^ frequency (mean ± SEM, *t* test, representative of 2 independent experiments). (I) Quantification of CD4^+^ GITR and OX40 frequency among iTreg cells (mean ± SEM, ANOVA, representative of 2–3 independent experiments). (J) WT and T^ΔAMPAR^ CD4 T cells derived from iTreg day 3 culture were co-cultured with CTV-labeled B6 WT CD8 T cells at varying ratios. Representative histograms of CTV by decreasing ratios of CD4:CD8 (left). Gating is of undivided CD8 T cells. Quantification of undivided CD8 T cell percentage by decreasing the ratio of CD4:CD8 (right). CTV = cell trace violet. (mean ± SEM, ANOVA, representative of 2 independent experiments). Statistical significance represented as ns > 0.05, ∗*p* < 0.05, ∗∗*p* < 0.01, ∗∗∗*p* < 0.001, ∗∗∗∗*p* < 0.0001.

Given that enhanced protection from EAE in T^ΔAMPAR^ mice was associated with reduced pro-inflammatory Th1 and Th17 cells and increased suppressive effector Tregs within the spinal cords of these mice, we next sought to evaluate whether T^ΔAMPAR^ deletion was associated with the altered differentiation of these cells *in vitro*. To this end, we isolated naive CD4 T cells from WT and T^ΔAMPAR^ mice and cultured them under Th1, Th17, and iTreg polarizing conditions for three days. Deletion of the AMPAR did not alter the expression of the canonical Th1 or Th17 transcription factors, T-bet and Rorγt, respectively (Figures 2A and 2B). Interestingly, however, we observed a significant increase in the frequency and geometric mean fluorescence intensity (gMFI) of Foxp3^+^ among T^ΔAMPAR^ iTregs (Figure 2C). Given that we have observed enhanced Foxp3 expression at day 3, we next wanted to evaluate the kinetics of Foxp3 expression over the course of iTreg differentiation. This analysis revealed enhanced Foxp3 expression was present as early as day 2 (Figure 2D). These data indicated that the AMPAR contributes to iTreg differentiation.

The differentiation of both iTreg and Th17 cells is reliant upon TGFβ; however, the presence of the pro-inflammatory cytokine, IL-6, at the time of differentiation biases polarization toward the Th17 subset.^29^ Because we observed enhanced iTreg differentiation under Treg differentiating conditions, we additionally sought to determine if this phenotype would be maintained even in the presence of IL-6. To this end, we cultured naive CD4 T cells in iTreg polarizing conditions and included a titration of IL6 to the culture. Indeed, we observed that T^ΔAMPAR^ iTregs maintained higher Foxp3 expression compared to WT CD4 T cells even in the presence of IL-6 (Figure 2E). Although we did not observe changes in Th17 differentiation based upon the expression of Rorγt, we sought to evaluate whether these cells, which also rely upon TGFβ for their differentiation, would have enhanced Foxp3 expression given the pronounced bias for Foxp3 generation observed in iTreg conditions as well as in the presence of IL-6. We observed that T^ΔAMPAR^ Th17 cells indeed contain higher frequencies of Foxp3^+^Rorγt^+^ cells (Figure 2F). These results revealed that T^ΔAMPAR^ CD4 T cells have a greater propensity to express Foxp3 even in suboptimal conditions.

Next, we assessed whether enhanced iTreg differentiation, known as peripheral Treg (pTreg) generation, occurred similarly *in vivo*. To this end, we adoptively transferred naive WT or T^ΔAMPAR^ CD4 T cells (CD62L^hi^, CD44^lo^, CD25^−^) into lymphopenic Rag2^−/−^ hosts and assessed their propensity for pTreg generation *in vivo*, as previously described.^30^^,^^31^^,^^32^ At week 3 post-transfer, host mice that received naive CD4 T cells from T^ΔAMPAR^ mice contained higher frequencies of CD4^+^Foxp3^+^ cells within mesenteric and peripheral lymph nodes relative to WT CD4 T cells (Figure 2G). While differential homeostatic proliferation could influence CD4^+^Foxp3^+^ cell generation, we observed no proliferation differences between WT and T^ΔAMPAR^ CD4 T cells (Figure S2A). These *in vivo* data corroborated our *in vitro* findings that T^ΔAMPAR^-deficient CD4 T cells experience enhanced iTreg differentiation.

Next, we evaluated whether the enhanced iTreg generation we observed *in vitro* and *in vivo* would be similarly found within the EAE model. To this end, we immunized WT and T^ΔAMPAR^ mice with myelin oligodendrocyte glycoprotein peptide 35–55 (MOG_35-55_) and restimulated dLN T cells *ex vivo* with MOG_35-55_ peptide under iTreg polarizing conditions. As expected, MOG_35-55_ restimulated dLN CD4 T cells from T^ΔAMPAR^ mice consistently had higher frequencies of CD4^+^Foxp3^+^ cells (Figure 2H). Taken together, the deletion of the AMPAR in CD4 T cells appears to drive the expression or stability of Foxp3, leading to enhanced iTreg differentiation.

While these findings suggest an enhanced capacity for iTreg cell differentiation in the absence of AMPAR signaling, we next assessed the functional phenotype of T^ΔAMPAR^ iTregs. Flow cytometric analysis revealed that T^ΔAMPAR^ iTregs expressed significantly higher levels of the canonical Treg activation markers GITR and OX40 (Figure 2I), consistent with transcriptional profiles from our scRNA-seq data (Figure 1J). To determine whether these cells retained immunosuppressive functionality, we performed an *in vitro* suppression assay. CD4^+^ T cells differentiated under iTreg-polarizing conditions from either WT or T^ΔAMPAR^ mice were co-cultured with CFSE-labeled C57BL/6J CD8^+^ Tconv responder cells at varying CD4:CD8 ratios. Notably, T^ΔAMPAR^ cells exhibited significantly greater suppressive activity at 1:2 and 1:4 CD4:CD8 ratios, as evidenced by the reduced proliferation of responder CD8 Tconv cells (Figure 2J). To further validate this finding, we also evaluated T^ΔAMPAR^ Treg function *in vivo*. To assess this, we performed an *in vivo* model of suppression where naive CD4 (CD62L^hi^, CD44^lo^) Tconv responder cells and natural Tregs (nTregs) (CD25^hi^) are co-adoptively transferred into a lymphopenic host, Rag2^−/−^ mice. These naive CD4 Tconv responder cells undergo homeostatic proliferation which can be suppressed by the presence of nTregs.^33^ We observed that T^ΔAMPAR^ nTregs reduced the expansion of naive Tconv cells significantly more than WT nTregs, indicating enhanced suppression by T^ΔAMPAR^ nTregs (Figure S2B). These results demonstrate that Tregs derived from AMPAR-deficient CD4^+^ T cells are not only generated at a higher frequency but also possess suppressive function, supporting a role for AMPAR signaling in restraining Treg differentiation and activity.

### AMPAR-deficient CD4 T cells changed cytokine sensitivity and metabolically shifted toward glycolysis and lipid metabolism

**Figure 3:**
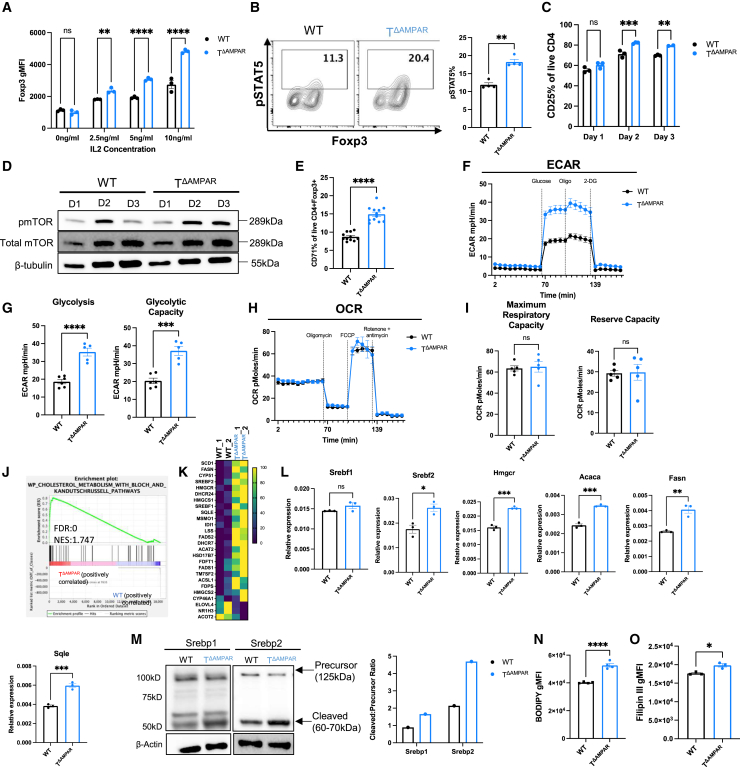
AMPAR-deficient CD4 T cells changed cytokine sensitivity and metabolically shifted toward glycolysis and lipid metabolism (A) Quantification of Foxp3 gMFI flow staining of WT and T^ΔAMPAR^ iTregs cultured under the titration of IL-2 (mean ± SEM, ANOVA, representative of 3 independent experiments). (B) Representative flow plot of pSTAT5^+^Foxp3^+^ expression (left) and quantification (right) from WT and T^ΔAMPAR^ iTreg cultures (mean ± SEM, *t* test, representative of 3 independent experiments). (C) Quantification of CD4^+^CD25^+^ frequency over the course of iTreg differentiation (mean ± SEM, ANOVA, representative of 3 independent experiments). (D) Western blot of WT and T^ΔAMPAR^ CD4 T cells collected on day 1, 2, and 3 of iTreg differentiation, immunoblotted for phosphorylated mTOR (pmTOR), total mTOR, and β-Actin expression (top to bottom, representative of 3 independent experiments). (E) Quantification of CD71^+^ cells among CD4^+^ T cells on day 3 of iTreg differentiation. (mean ± SEM, *t* test, representative of 3 independent experiments). (F–I) Seahorse metabolic flux analysis of iTreg cells. (F) extracellular acidification rate (ECAR) measurement over time (minutes) and (G) quantification of glycolysis (left) and glycolytic capacity (right) measurements. (H) oxygen consumption rate (OCR) measurement over time (minutes) and (I) quantification of maximum respiratory capacity (left) and reserve capacity (right) measurements (mean ± SEM, ANOVA, representative of 3 independent experiments). (J and K) WT and T^ΔAMPAR^ CD4 were cultured under iTreg polarizing conditions and subjected to bulk RNA sequencing and analysis. (J) GSEA plot of cholesterol metabolism pathway enrichment in T^ΔAMPAR^ versus WT cells. (K) Heatmap of genes associated with cholesterol metabolism in T^ΔAMPAR^ versus WT. (L) Quantification of *Srebf1*, *Srebf2*, *Hmgcr*, *Acaca*, *Fasn*, and *Sqle* mRNA relative expression in WT and T^ΔAMPAR^ CD4 from iTreg culture determined by qPCR (mean ± SEM, ANOVA, representative of 2 independent experiments). (M) Western blot images (left) of WT and T^ΔAMPAR^ immunoblotted for Srebf1 and Srebf2 (image is representative of 2 independent experiments) and quantification (right) of cleaved to precursor ratios analyzed by densitometry. (N and O) Cell staining for Lipid and cholesterol content. (N) Bodipy and (O) Filipin III gMFI flow staining of WT and T^ΔAMPAR^ CD4 from day 2 iTreg cultures (mean ± SEM, *t* test, representative of 3 independent experiments). Statistical significance represented as ns > 0.05, ∗*p* < 0.05, ∗∗*p* < 0.01, ∗∗∗*p* < 0.001, ∗∗∗∗*p* < 0.0001.

Because of the clear role of the AMPAR in regulating iTreg differentiation and function, we next sought to assess the mechanistic basis for this phenotype. As both TGFβ and IL-2 cytokine signaling are known to positively regulate *Foxp3* transcription, and our scRNAseq data showed enhanced TGFβ and IL-2 signaling in T^ΔAMPAR^ Tregs (Figure 1H), we next assessed if the differential downstream signaling of these cytokines was involved in the enhanced Foxp3 expression observed in our *in vitro* system.^34^^,^^35^ To this end, we differentiated WT and T^ΔAMPAR^ iTregs under a titration of TGFβ and IL-2 and evaluated Foxp3 expression. Interestingly, in both contexts, we observed that even at limiting conditions of TGFβ and IL-2, T^ΔAMPAR^ iTregs maintained higher expression of Foxp3 compared to WT iTregs (Figure 3A; Figure S2C). Thus, AMPAR deficiency is associated with increased sensitivity to TGFβ and IL-2. To determine if this enhanced sensitivity was due to enhanced signaling through these cytokine receptors, we evaluated whether there were changes to the phosphorylation state of their canonical downstream signal transducers. In the assessment of the major positive signaling transducer downstream of receptors of TGFβ receptors, SMAD3, we did not observe a significant difference (Figure S2D). However, we observed that the phosphorylation of the primary signal transducer of IL-2 signaling, STAT5, was significantly increased among T^ΔAMPAR^ cells (Figure 3B). These data suggested that the sensitivity of T^ΔAMPAR^ CD4 to IL-2 was associated with increased signaling. Consistent with these findings, the expression of the high affinity IL-2 receptor (CD25) was also increased in T^ΔAMPAR^ CD4 T cells over the course of iTreg differentiation (Figure 3C). Altogether, AMPAR deficiency in CD4 T cells leads to enhanced TGFβ and IL-2 cytokine sensitivity and signaling, particularly for IL-2, which supports both the differentiation and function of T^ΔAMPAR^ iTregs.^36^^,^^37^^,^^38^

This observed increase in IL-2 sensitivity and signaling in T^ΔAMPAR^ CD4 T cells prompted us to hypothesize that the enhanced differentiation of these cells may be dependent upon the mammalian target of rapamycin complex 1 (mTORC1) pathway, a known master metabolic regulator of T cell activation, proliferation, and differentiation.^39^ Previous work has demonstrated that IL-2 regulates mTORC1 and that mTORC1 in turn supports the effector functions of Tregs.^22^^,^^23^^,^^24^^,^^40^ Given that our scRNAseq dataset showed increased expression of mTORC1 and glycolysis pathway genes among spinal cord infiltrating T^ΔAMPAR^ Tregs (Figure 1I), we next assessed this pathway at the protein level in cells derived from iTreg polarizing culture conditions. Our assessment of this pathway throughout differentiation revealed that although both WT and T^ΔAMPAR^ CD4 initially exhibit similar levels of phosphorylated or activated mTOR (pmTOR), this activation is sustained in T^ΔAMPAR^ CD4 T cells through the completion of differentiation (Figure 3D). Consistent with increased mTORC1 activation, the expression of CD71, an mTOR-dependent cell surface transferrin receptor, was significantly increased in T^ΔAMPAR^ CD4 T cells (Figure 3E).^41^ These data suggested that mTORC1 signaling is enhanced in T^ΔAMPAR^ CD4 cells undergoing iTreg differentiation.

Because mTORC1 activity is tightly linked to cellular metabolism, we next sought to evaluate glycolysis and mitochondrial respiration in T^ΔAMPAR^ cells derived from iTreg culture conditions using a seahorse assay.^42^ We observed a significant increase in the extracellular acidification rate (ECAR) in T^ΔAMPAR^ cells derived from iTreg cultures subjected to a glucose stress test, indicating enhanced glycolysis and glycolytic capacity (Figures 3F and 3G). We did not observe a significant alteration to the oxidative consumption rate (OCR) of T^ΔAMPAR^ cells undergoing a mitochondrial stress test (Figures 3H and 3I). These data suggest that T^ΔAMPAR^ CD4 cells undergo a pronounced shift toward mTORC1 pathway upregulation and increased glycolytic metabolism, likely to support their effector functional state during iTreg differentiation.

Next, we wanted to evaluate if these pathways were transcriptionally regulated and if they contributed to T^ΔAMPAR^ iTreg differentiation. To evaluate this, we chose to perform bulk RNA sequencing (RNAseq) analysis of WT and T^ΔAMPAR^ CD4 T cells cultured under the iTreg differentiation condition. At day 3 of iTreg differentiation, we did not observe mTORC1 or glycolysis pathway changes at the transcriptional level (data not shown). Interestingly, this analysis revealed that an additional metabolic pathway, cholesterol synthesis, was significantly enriched in T^ΔAMPAR^ CD4 (Figures 3J and 3K). Lipid metabolism, which includes fatty acid and cholesterol synthesis, has been shown to play a positive role in the differentiation and function of Tregs.^43^^,^^44^^,^^45^ Furthermore, mTORC1 signaling has previously been associated with the upregulation of the cholesterol synthesis pathway in Tregs.^24^ Upon further analysis, the expression of genes specific to the cholesterol synthesis pathway, including *Srebf2*, *Acaca*, *Fasn*, *Hmgcr*, and *Sqle,* were all significantly increased among T^ΔAMPAR^ cells derived from iTreg culture conditions (Figure 3L). Given the changes we detected at the RNA level, we next sought to evaluate if there were notable changes to lipid metabolism pathways at the protein level. To this end, we evaluated the enzymatic activity of sterol regulatory element binding proteins (Srebp) 1 and 2, which are required for fatty acid and cholesterol synthesis, respectively. T^ΔAMPAR^ CD4 cells derived from iTreg cultures exhibited increased levels of the enzymatically active cleavage product for both Srebp1 and Srebp2 (Figure 3M). Because both RNA and protein levels indicated that the fatty acid and cholesterol pathways were upregulated, we then sought to determine if this resulted in a quantifiable change to fatty acid and cholesterol content within T^ΔAMPAR^ cells from iTreg conditions. To evaluate this, we utilized two cell-permeable dyes: Bodipy to quantify fatty acid content and Filipin III to quantify cholesterol content. Interestingly, we observed a significant increase in both the fatty acid and cholesterol content measured by increased gMFI of these respective dyes (Figures 3N and 3O). To confirm these findings, we additionally performed untargeted metabolomics of T^ΔAMPAR^ CD4 cells under iTreg polarizing conditions. We found that metabolites associated with the glycerophospholipid synthesis pathway were one of the top differentially represented KEGG pathways (Figure S3A). Altogether, these data show that fatty acid and cholesterol synthesis are significantly increased in T^ΔAMPAR^ CD4 cells, contributing to iTreg differentiation and function.

### AMPAR antagonists may provide therapeutic benefit

**Figure 4:**
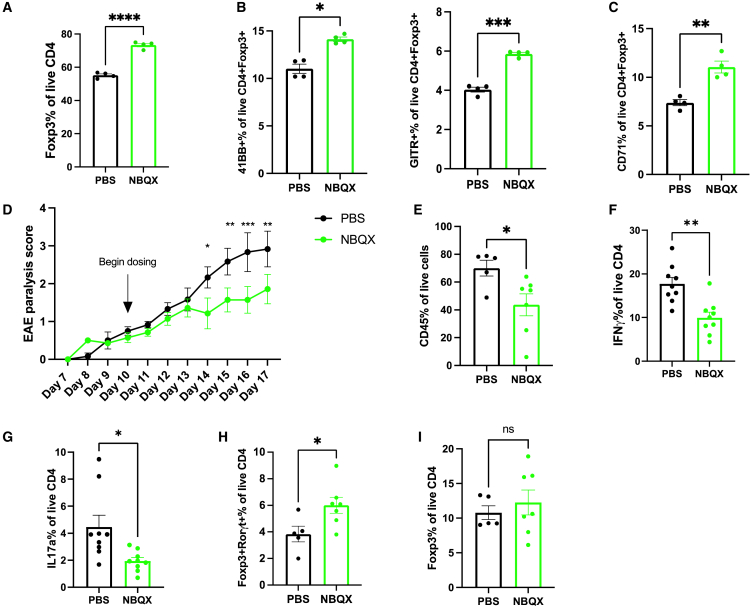
AMPA receptor antagonists enhance Treg generation and provide protection from severe autoimmune disease (A–C) Naive CD4 T cells were isolated from B6 WT mice and cultured *in vitro* under iTreg polarizing conditions in the presence of PBS (vehicle) or 50 μM of NBQX. Cells were analyzed on day 3. (A) Quantification of CD4^+^Foxp3^+^ frequency. (B and C) Quantification of 41BB, GITR (B), and CD71 (C) frequency among CD4^+^Foxp3^+^ cells (mean ± SEM, ANOVA, representative of 3 independent experiments with 3–4 biological replicates per group). (D–G) B6 WT mice were immunized with CFA-MOG and injected with PTX to induce EAE. Disease monitoring began on day 7, and mice were separated into PBS and NBQX treatment groups. On day 10, treatment began with the q12 h dosing of PBS or NBQX (30 mg/kg) daily until day 17. (D) EAE paralysis scores over the course of disease in PBS and NBQX-treated mice. Quantification of (E) CD45 proportion of spinal cord infiltrates, (F) IFN*γ*, (G) IL17, (H) Foxp3^+^Rorgt^+^, and (I) Foxp3^+^ proportion among live CD4 cells from the spinal cords of PBS and NBQX-treated mice (mean ± SEM, ANOVA, representative of 3 independent experiments with 5–9 biological replicates per group). Statistical significance represented as ns > 0.05, ∗*p* < 0.05, ∗∗*p* < 0.01, ∗∗∗*p* < 0.001, ∗∗∗∗*p* < 0.0001.

Our findings have underscored the therapeutic potential of targeting T cell derived AMPARs to promote immunosuppression. Therefore, we next sought to assess whether the pharmacological inhibition of the AMPAR would similarly enhance Treg generation and protect mice from severe autoimmune disease. To this end, we first sought to evaluate if the polarization of B6 WT naive CD4 T cells to iTreg in the presence of a competitive AMPAR antagonist, NBQX, would affect differentiation. The addition of NBQX to B6 WT iTreg culturing conditions significantly increased the frequency of CD4^+^Foxp3^+^ cells (Figure 4A). Interestingly, when we further evaluated NBQX-treated CD4 T cells, we noted a significant increase in the expression of both 4-1BB and GITR effector molecules (Figure 4B). Additionally, NBQX-treated iTregs also had higher surface expression of CD71, suggesting enhanced mTORC1 activity (Figure 4C). These data supported the observation of enhanced T^ΔAMPAR^ iTreg differentiation and effector molecule expression, underscoring the anti-inflammatory potential of AMPAR blockade.

Next, we evaluated the effect of an AMPAR antagonist on EAE progression. To assess the therapeutic utility of AMPAR targeting in EAE, we began treatment with NBQX after EAE was established. Interestingly, NBQX treatment significantly reduced EAE disease scores and protected mice from developing severe paralysis (Figure 4D). Notably, these findings are supported by previous studies, which similarly found that NBQX treatment protected rats from developing severe EAE.^14^ However, this previous study did not evaluate the immune cell compartment and its contribution to autoimmune disease protection. Upon the evaluation of this compartment, we noted fewer total immune cells were present within the NBQX-treated spinal cords at the peak of disease (Figure 4E). Additionally, IFNγ and IL17 inflammatory cytokine expression was significantly reduced among NBQX-treated spinal cord infiltrating CD4 T cells (Figures 4F and 4G). Similar to previous findings from *in vitro* T^ΔAMPAR^ Th17 differentiation (Figure 2F), the infiltrating Foxp3^+^Rorγt^+^ CD4 population was also significantly enriched in spinal cords from NBQX-treated mice (Figure 4H). While not significant, we also observed a trend of increased Foxp3^+^ cells among spinal cord infiltrating CD4 cells from these mice (Figure 4I). Because the dosing regimen used in this experiment began *after* the disease was established, it is possible that this difference in timing may be responsible for the smaller effect relative to the genetic T cell-specific deletion of the AMPAR model. However, these data offer a preliminary indication that targeting the AMPAR in the context of autoimmunity provides therapeutic benefit, in part, by its effect on CD4 T cells.

## Discussion

Herein, we characterized a novel role of the AMPAR in regulating CD4 T cell skewing to an immunosuppressive phenotype. Specifically, we found that AMPAR deletion in T cells protected mice from severe experimental autoimmune encephalomyelitis (EAE), and this protection was associated with an enhanced effector Treg presence. We observed that AMPAR deficiency intrinsically polarized CD4 cells toward iTreg differentiation both *in vitro* and *in vivo*. AMPAR-deficient CD4 T cells exhibited increased sensitivity to IL-2 and activation of STAT5, along with enhanced mTORC1 activity, glycolysis, and lipid metabolism—key processes contributing to Treg differentiation and suppressive function.^24^^,^^46^

Glutamate is a key metabolite produced by proliferating cells and accumulates at sites of inflammation.^47^^,^^48^^,^^49^ Metabolites play a diverse and active role in coordinating tissue responses, acting as both metabolic substrates and signaling molecules that can regulate inflammation, regeneration, immunity, and repair.^50^^,^^51^ Whether the elevated level of glutamate in the inflammatory environment may have any impact on the T cell response trajectory during inflammation is an open question.

Glutamate is the primary ligand for the ionotropic AMPAR, which plays a key role in neuronal communication. Although the AMPAR is well-characterized in the nervous system, its role in T cells remains unknown. Previous work has shown that the pharmacological inhibition of the AMPAR correlated with the reduced inflammatory state or activation of T cells.^10^^,^^12^^,^^52^^,^^53^ However, these observations were largely correlative and lacked direct mechanistic insight into AMPAR function within T cells.

Our study revealed a previously unrecognized role for the AMPAR in regulating Treg differentiation and function, thereby advancing our mechanistic understanding of the anti-inflammatory effects mediated by AMPAR blockade. These insights support our hypothesis that T cell derived ionotropic AMPA receptors serve as environmental sensors of inflammation by integrating metabolite balances, such as glutamate, to coordinate the adaptive immune response. Inhibition or loss of the AMPA receptor thereby removes the brakes on Treg differentiation, which ultimately promotes immune suppression and resolution. Therefore, AMPA receptors emerge as key regulators of the immune response, linking metabolic signals to T cell fate and function.

The AMPAR is a ligand-gated ion channel that can flux Na^+^, K^+^, and Ca^2+^. Ion channels influence cellular metabolism through multiple mechanisms, including modulating membrane potential, alterations in ionic homeostasis, and ATP consumption associated with active ion transport.^54^^,^^55^^,^^56^^,^^57^^,^^58^ Beyond its canonical ionotropic functions, AMPAR may also regulate metabolism in an ion flux-independent manner. Studies in neurons have shown that the ionotropic AMPAR has “metabotropic” properties, activating ERK signaling in a PI3K/Akt dependent manner or via interaction with Lyn.^59^^,^^60^ Our findings demonstrate that AMPAR deficiency in CD4^+^ T cells leads to distinct metabolic reprogramming, underscoring a critical link between ion channel signaling and immune cell metabolism. These results position the AMPAR as a potential integrator of environmental cues and intracellular metabolic programming in T cells and highlight its potential as a therapeutic target in autoimmune disease.

Taken together, our concordant *in vitro* and *in vivo* findings strongly support the conclusion that AMPAR signaling modulates T cell metabolic programming, inhibits Treg differentiation, and ultimately contributes to the development of severe autoimmune disease.

### Limitations of the study

Although we observed a pronounced increase in Treg cell frequency among spinal cord infiltrates in the EAE model and focused our investigation on the AMPAR’s role in CD4 T cell metabolism and Treg differentiation, we cannot exclude the possibility that AMPAR-deficient CD4^+^ Tconv cells also exhibit subtle phenotypic changes that contribute to the observed protection from severe autoimmune disease. To further evaluate and strengthen our understanding of the AMPAR and its function in Treg cells, we may utilize a Foxp3-Cre model for future studies. This model may allow us to understand not only the impact of the AMPA receptor on Treg function but the relative role of these AMPAR deficient Treg cells in disease models such as EAE.

## Resource availability

### Lead contact

Further information and requests for resources and reagents should be directed to and will be fulfilled by the lead contact, Hong Yu (hyu13@jhmi.edu; yuh5@uthscsa.edu).

### Materials availability

This study did not generate any unique reagents.

### Data and code availability

The bulk RNASeq and scRNASeq data are openly available in Gene Expression Omnibus NCBI (GEO) at https://www.ncbi.nlm.nih.gov/gds with the accession numbers GSE275380 and GSE275381. This article does not report original code. The original western blot images have been deposited at Mendeley Data: https://data.mendeley.com/datasets/vdd88gxcm6/1. Any additional information required to reanalyze the data reported in this article is available from the lead contact upon request.

## Acknowledgments

The authors would like to thank Dr. Rachel Helms and Dr. Franck Housseau for their constructive discussion of the project and editing of the article. We wish to thank Erin McCadden for her assistance in laboratory management. We would also like to acknowledge Camille Jaime for her assistance in creating the graphical abstract. The graphical abstract was created in BioRender: Mitchell-Flack, M. (2025) https://BioRender.com/xsgw5h3. This work was supported by the Bloomberg-Kimmel Institute for Cancer Immunotherapy (BKI) fund at the 10.13039/100012304Johns Hopkins School of Medicine, United States.

## Author contributions

M.M-F.: conceptualization, data curation, formal analysis, investigation, methodology, validation, visualization, writing – original draft, and writing - review and editing. M.H., Y.Z., H.N., S.S.M., and A.S.: investigation. A.T. and R.B.: methodology and investigation. H.G.: conceptualization, methodology, and investigation. B.L.: conceptualization. C.C. and M.P.: formal analysis, methodology, and software. R.H.: conceptualization, resources, and supervision. H.Y.: conceptualization, data curation, formal analysis, investigation, methodology, supervision, validation, and writing – reviewing and editing. D.P.: conceptualization, funding acquisition, project administration, resources, supervision, validation, and writing – reviewing and editing.

## Declaration of interests

The authors declare no competing interests.

## STAR★Methods

### Key resources table

**Table:** 

REAGENT or RESOURCE	SOURCE	IDENTIFIER
**Antibodies**		

Anti-CD45 Brilliant Violet 650	Invitrogen	Cat# 416-0451-82
Anti-CD4 APC/Cyanine7	BioLegend	Cat# 100414; RRID: AB_312699
Ant-CD8 PerCP/Cyanine5.5	BioLegend	Cat# 140418; RRID: AB_2800651
Anti-TNFa Alexa Fluor 700	BioLegend	Cat# 506338; RRID: AB_2562918
Anti-GM-CSF APC	BioLegend	Cat# 505414; RRID: AB_2721461
Anti-IFNg Brilliant Violet 711	BioLegend	Cat# 505836; RRID: AB_2650928
Anti-IL17 PE/Cyanine7	BioLegend	Cat# 506922; RRID: AB_2125011
Anti-CD25 Brilliant Violet 605	BioLegend	Cat# 102036; RRID: AB_2563059
Anti-CD44 Alexa Fluor 700	BD Biosciences	Cat# 560567; RRID: AB_17227480
Anti-CD45RB FITC	BioLegend	Cat# 103306; RRID: AB_313013
Anti-CD62L Brilliant Violet 421	BioLegend	Cat# 104436; RRID: AB_2562560
Anti-CD69 APC-Cy7	BD Biosciences	Cat# 561240, RRID: AB_10611852
Anti-CD71 PE/Dazzle 594	BioLegend	Cat# 113818; RRID: AB_2749883
Anti-CD134 (OX-40) PE/Cyanine7	BioLegend	Cat# 119416; RRID: AB_2566155
Anti-CD137 (4-1BB) PE	Invitrogen	Cat# 17-1371-82; RRID: AB_2573162
Anti-CD357 (GITR) APC	BioLegend	Cat# 126312; RRID: AB_2271858
Anti-Tbet PE/Dazzle 594	BioLegend	Cat# 644828; RRID: AB_2565677
Anti-RorgT PE	BD Biosciences	Cat# 562607; RRID: AB_11153137
Anti-Foxp3 Alexa Fluor 488	Invitrogene	Cat# 53-5773-82; RRID: AB_763537
Anti-pSTAT5 (pY694)	BD Biosciences	Cat# 612567; RRID: AB_399858
Anti-phospho-mTOR (Ser2448)	Cell Signaling	Cat# 2971; RRID: AB_330970
Anti-mTOR	Cell Signaling	Cat# 2983; RRID: AB_2105622
Anti-pSMAD3	Cell Signaling	Cat# 9520; RRID: AB_2193207
Anti-SMAD2/3	Cell Signaling	Cat# 3102; RRID: AB_10698742
Anti-Srebp1	Abcam	Cat# ab3259; RRID: AB_303650
Anti-Srebp2	Abcam	Cat# ab32682; RRID: AB_779079
Anti-b-tubulin	Cell Signaling	Cat# 86298; RRID: AB_2715541
Anti-β-actin	Cell Signaling	Cat# 4970; RRID: 2223172
HRP-conjugated anti-mouse	Cell Signaling	Cat# 7076; RRID: AB_330924
HRP-conjugated anti-rabbit	Cell Signaling	Cat# 7074; RRID: AB_2099233
Anti-CD3e purified	Bio-X-Cell	Cat# BE0001-1;RRID: AB_1107634
Anti-CD28 purified	Bio-X-Cell	Cat# BE0015-5; RRID: AB_1107628
Anti-IL4 purified	Bio-X-Cell	Cat# BE0045, RRID: AB_1107707
Anti-IFNg purified	Bio-X-Cell	Cat# BE0055; RRID: AB_1107694

**Chemicals, peptides, and recombinant proteins**		

Mouse IL2	PeproTech	Cat# 212-12
Mouse IL-12p70	PeproTech	Cat# 210-12
Mouse IFNg	PeproTech	Cat# 315-05
Human TGFb	PeproTech	Cat# 100-21
Mouse IL6	PeproTech	Cat# 216-15
Mouse IL2	PeproTech	Cat# 212-12
BODIPY^TM^ 493/503	ThermoFisher	Cat# D3922
Filipin III	Sigma	Cat# SAE0087
Oligomycin	Sigma	Cat# 495455
Carbonyl cyanide 4-(trifluoromethoxy)phenylhydrazone (FCCP)	Sigma	Cat# SML2959
Rotenone	Sigma	Cat# 45656
Antimycin	Sigma	Cat# A8674
2-Deoxy-D-glucose (2-DG)	Sigma	Cat# D8375
PMA	Sigma	Cat# P1585
Ionomycin	Sigma	Cat# 19657
GolgiPlug	BD Biosciences	Cat# 555029
Cell counting bead	Spherotech	Cat# pp-50-10
CellTrace violet dye	Invitrogen	Cat# C34571
RIPA buffer	Sigma	Cat# RO278
Protease inhibitor cocktail	ThermoFisher	Cat# 78440
4-15% Mini-Protean TGX SDS-PAGE gels	Biorad	Cat# 4561085

**Critical commercial assays**		

EAE Induction Kits	Hooke	Cat# EK-2110
eBioscience™ Foxp3/Transcription Factor Staining Buffer Set	Invitrogen	Cat# 00-5523-00
Live/Dead Fixable Aqua Dead Cell Stain Kit	Invitrogen	Cat# L34957
Live/Dead Fixable near-IR Dead Cell Stain Kit	Invitrogen	Cat# L34975
Naive CD4^+^ T cell Isolation Kit, mouse	Miltenyi Biotec	Cat# 130-104-453
Total CD4^+^ T cell Isolation Kit, mouse	Miltenyi Biotec	Cat# 130-104-454
CD4^+^CD25^+^ Regulatory T cell Isolation Kit, mouse	Miltenyi Biotec	Cat# 130-091-041
CD8 T cell Isolation Kit, mouse	Miltenyi Biotec	Cat# 130-117-044
Dynabead mouse-T activator CD3/28 bead	Gibco	Cat# 11456D
RNeasy Micro Kit	Qiagen	Cat# 70004
High-Capacity cDNA Reverse Transcription Kit	ThermoFisher	Cat# 4368814
PowerUp SYBR Green Master Mix	ThermoFisher	Cat# A25780
Pierce BCA assay	ThermoFisher	Cat# 23225
PVDF membrane	Biorad	Cat# 1620177
SuperSignal ECL west femto	ThermoFisher	Cat# 34095

**Deposited data**		

Bulk RNA-seq data	Original data	GEO GSE275380
scRNA-seq data	Original data	GSE275381

**Experimental models: Organisms/strains**		

Mouse: CD4Cre	The Jackson Laboratory	strain# 022071
Mouse: Rag2KO	The Jackson Laboratory	strain# 008449
Mouse: B6 CD45.1	The Jackson Laboratory	Strain# 002014
Mouse: C57Bl/6J	The Jackson Laboratory	Strain# 000664
Mouse: GluA1 *fl/*fl, GluA2 *fl/fl*, and GluA3 *fl/fl*	Richard Huganir	N/A

**Oligonucleotides**		

Gria2 Forward: TAC GAG TGG CAC ACT GAG GA	This paper	N/A
Gria2 Reverse: CCC AGA GAG AGA TCT TGG CGA	This paper	N/A
Gria3 Forward: AAG AAC CTC GTG ACC CAC AA	This paper	N/A
Gria3 Reverse: CGC CCA GAA AGT GAT CTT GGA G	This paper	N/A
*Srebf1*Forward: GCAGCCACCATCTA GCCTG	This paper	N/A
*Srebf1*Reverse: CCCCATGACTAAGTCC TTCAACT	This paper	N/A
*Srebf2* Forward: GCAGCAACGGGA CCATTCT	This paper	N/A
*Srebf2* Reverse: CCCCATGACTAAGTC CTTCAACT	This paper	N/A
*Hmgcr* Forward: AGCTTGCCCGAATT GTATGTG	This paper	N/A
*Hmgcr* Reverse: TCTGTTGTGAACCAT GTGACTTC	This paper	N/A
*Acaca* Forward: ATGGGCGGAATGGT CTCTTTC	This paper	N/A
*Acaca* Reverse: TGGGGACCTTGTCT TCATCAT	This paper	N/A
*Fasn* Forward: GGAGGTGGTGATAG CCGGTAT	This paper	N/A
*Fasn* Reverse: TGGGTAATCCATAGA GCCCAG	This paper	N/A
*Sqle* Forward: ATAAGAAATGCGG GGATGTCAC	This paper	N/A
*Sqle* Reverse: ATATCCGAGAAGG CAGCGAAC	This paper	N/A

**Software and algorithms**		

Prism	Prism	Prism
FlowJo	FlowJo™ v10.8 software	Becton, Dickinson and Company
ImageJ	ImageJ^61^	https://doi.org/10.1038/nmeth.2089
BioRender	Created in BioRender. Mitchell-flack, M. (2025) https://BioRender.com/xsgw5h3	https://biorender.com

### Experimental model and study participant details

#### Mice

GluA1 *fl*/fl, GluA2 *fl*/*fl*, and GluA3 *fl/fl* mice were generated as previously described and generously provided by Dr. Richard Huganir.^17^^,^^19^^,^^62^ Homozygous GluA1/2/3 *fL/fL* CD4Cre negative littermate control mice are referred to as wild-type (WT). Homozygous GluA1/2/3 *fL/fL* CD4Cre positive mice are referred to as T^ΔAMPAR^ mice. CD4 Cre recombinase (strain# 022071), Rag2^−/−^ (strain# 008449), B6 WT CD45.1 (strain# 002014), and C57Bl/6J (B6 WT) (strain# 000664) mice were obtained from the Jackson Laboratory and maintained by breeding in house. Both male and female mice were used in all experiments, and no significant sex-associated differences were observed. All mouse colonies were maintained in accordance with the guidelines of Johns Hopkins University and the institutional animal care and use committee.

### Method details

#### EAE induction, scoring, and assessment

EAE was induced in nine to twelve weeks old female mice by subcutaneous injection of 200 μg MOG_35–55_ peptide (100 μg/flank) emulsified in Complete Freund’s Adjuvant (CFA) into the flanks at two different sites (Hooke Laboratories, EK-2110). In addition, the mice received 200 ng pertussis toxin (PTX) (Hooke Laboratories, EK-2110) intraperitoneally at the time of immunization and 24 h later. For AMPAR inhibitor EAE studies, beginning on day 10 mice were dosed twice daily every 12 h with 30 mg/kg NBQX or PBS. Body weight was measured daily beginning on day 7 until the day of harvest. Clinical signs of EAE were assessed daily beginning on day 7 until end of experiment according to a 5-point scale (Hooke Laboratories, EK-2110): no obvious changes in motor function, 0; tip of tail is limp, 0.5; limp tail, 1; limp tail plus hindlimb inhibition, 1.5; limp tail and weakness of hind limbs, 2; limp tail and dragging of hind limbs (legs have some movement but both are dragging at the feet), 2.5; limp tail and complete paralysis of hind limbs, 3; limp tail and complete paralysis of hind limbs and mouse has difficulty turning over, 3.5; limp tail, complete hindlimb, and partial front limb paralysis, 4; complete hind and partial front limb paralysis, no movement around cage, 4.5; moribund or death due to paralysis, 5. At the time of harvest, draining lymph nodes were isolated and homogenized to single-cell suspensions and analyzed according to flow cytometry methods. Spinal cords were isolated and a Percoll (Sigma, P1644) gradient was performed to enrich for leukocytes as described previously and analyzed according to flow cytometry methods. For single cell RNA sequencing, spinal cords were isolated (5 mice/group) and a Percoll (Sigma, P1644) gradient was performed to enrich for leukocytes. Enriched leukocytes were pooled by group and stained for by Live/Dead Fixable Aqua Dead Cell Stain Kit (Invitrogen, L34957) and anti-CD4 (RM4-5). Pooled samples were then sorted by FACS on live CD4^+^ positive populations. Cells were washed and resuspended in complete RPMI and submitted to the Johns Hopkins Single Cell & Transcriptomics Core for processing and sequencing.

#### Single cell RNA sequencing (scRNAseq)

Cell counts and viability were determined using the Cell Countess 3 with Trypan Blue. A maximum volume of 86.4 μL/sample was used for processing to target up to 20,000 cells. Cells were combined with RT reagents and loaded onto 10X Next GEM Chip M along with 3′ HT gel beads. The NextGEM protocol was run on the 10X Chromium X to create GEMs (gel bead in emulsion), composed of a single cell, gel bead with unique barcode and UMI primer, and RT reagents. 180 uL of emulsion is retrieved from the chip, split into 2 wells, and incubated (45 min at 53C, 5 min at 85C, cool to 4C), generating barcoded cDNA from each cell. The GEMs are broken using Recovery Agent and cDNA is cleaned, following manufacturer’s instructions using MyOne SILANE beads. cDNA is amplified for 11 cycles (3 min @ 98°C, 11 cycle: 15 s @98°C, 20 s @63°C, 1 min @72°C; 1 min @ 72°C, cool to 4°C). Samples are cleaned using 0.6X SPRIselect beads. QC is completed using Qubit and Bioanalyzer to determine size and concentrations. 10 uL of amplified cDNA is carried into library prep. Fragmentation, end repair and A-tailing are completed (5 min @ 32°C, 30 min @ 65°C, cool to 4°C), and samples are cleaned up using double sided size selection (0.6X, 0.8X) with SPRIselect beads. Adaptor ligation (15 min @ 20°C, cool to 4°C), 0.8X cleanup and amplification are performed, with PCR using unique i7 index sequences. Libraries undergo a final cleanup using double sided size selection (0.6X, 0.8X) with SPRIselect beads. Library QC is performed using Qubit, Bioanalyzer and KAPA library quantification qPCR kit. Libraries are sequenced on the Illumina NovaSeq 6000 using v1.5 kits, targeting 50K reads/cell, at read lengths of 28 (R1), 8 (i7), 91 (R2). Demultiplexing and FASTQ generation is completed using Illumina’s BaseSpace software. Sample preparation and sequencing completed by Dr. Tyler J. Creamer and Linda Orzolek of the Johns Hopkins Single Cell & Transcriptomics Core.

#### scRNAseq analysis

Fastq files were processed using Cellranger version 7.0.0. In addition to the automated filtering in Cellranger, cells were further filtered on UMI (>500), number of expressed genes (>25), and contribution of mitochondrial RNA to reads (<25%). Seurat v4.3.0^63^ was use for normalization, variable features selection, scaling, principal component analysis, UMAP^64^ and clustering. Sets of genes associated with technical artifact or variance were removed prior to all analyses. Clusters of contaminant cells that passed filter were selected and removed prior to re-analysis of the data. Mann-Whitney U test was used for differential expression. fgsea v1.24.0^65^ was used for gene set enrichment analysis using -log(p) ∗ sign(fc) as the ranking metric with gene sets selected from the Molecular Signatures Database.^66^ Running enrichment plots show the true data (blue line) together with 100 random permutations of the gene ranks (gray lines) representing the null distribution.

#### Cell isolation and *in vitro* culture

For homeostasis studies, thymus, lymph node, and spleen were isolated from mice and single cell suspensions were prepared prior to flow cytometry staining and analysis. Naive CD4 cells were isolated from the spleen and peripheral lymph node by a magnetic bead-based purification according to the manufacturer’s instructions (Miltenyi Biotech, 130-104-453). Purified naive CD4 cells were stimulated with plate-bound 5 μg/ml anti-CD3ε (Bio-X-Cell, BE0001-1) and soluble 2 μg/ml anti-CD28 (Bio-X-Cell, BE0015-5) for 3 days, in RPMI1640 medium (Gibco, 11875093) supplemented with 10%FBS, HEPES, penicillin/streptomycin, MEM Non-Essential Amino Acids, and β-mercaptoethanol unless otherwise noted. For AMPAR inhibitor *in vitro* studies, cells were cultured in the presence of 50 μM NBQX or solvent control PBS. For *ex vivo* MOG_35-55_ restimulation, draining lymph node cells were plated with 50 μg/mL MOG_35-55_ peptide (protocol adapted from^67^). For Th1 cell differentiation, cells were stimulated in the presence of 10 ng/mL mouse IL2 (PeproTech, 212-12), 10 ng/mL mouse IL-12p70 (PeproTech, 210-12), 10 ng/mL mouse IFNγ (Peprotech, 315-05), 5 μg/ml anti-IL4 antibody (Bio X Cell, BE0045). For Th17 cell differentiation, cells were stimulated in the presence of 5 ng/mL TGFβ (PeproTech, 100-21), 20 ng/mL IL6 (PeproTech, 216-16), 5 μg/mL anti-IFNγ antibody (Bio X Cell, BE0055), and 5 μg/mL anti-IL4 antibody (Bio X Cell, BE0045). For iTreg cell differentiation, cells were stimulated in the presence of 10 ng/mL mouse IL2, 10 ng/mL human TGFβ (PeproTech, 100-21), 10 μg/mL anti-IL4 antibody, and 10 μg/mL anti-IFNγ antibody.

#### Quantitative RT-PCR

RNA was isolated using the RNeasy Micro Kit (Qiagen, 70004) following the manufacturer’s instructions. RNA was converted to cDNA using the High-Capacity cDNA Reverse Transcription Kit (ThermoFisher Scientific, 4368814) according to the manufacturer’s instructions. The primers of murine genes were purchased from Integrated DNA Technology (IDT). qPCR was performed using the PowerUp SYBR Green Master Mix (ThermoFisher Scientific, A25780) and the Applied Biosystems StepOnePlus 96-well real-time PCR system. ΔΔCt values were normalized to βactin RNA, and the relative quantification of gene expression ratio is shown in comparison with the control. Primers used for *Gria2* were: TAC GAG TGG CAC ACT GAG GA (forward); CCC AGA GAG AGA TCT TGG CGA (reverse) and for *Gria3* were: AAG AAC CTC GTG ACC CAC AA (forward); CGC CCA GAA AGT GAT CTT GGA G (reverse). Primers for the mevalonate pathway include: *Srebf1*; GCAGCCACCATCTAGCCTG (forward) and CAGCAGTGAGTCTGCCTTGAT (reverse), *Srebf2*; GCAGCAACGGGACCATTCT (forward) and CCCCATGACTAAGTCCTTCAACT (reverse), *Hmgcr*; AGCTTGCCCGAATTGTATGTG (forward) and TCTGTTGTGAACCATGTGACTTC (reverse), *Acaca*; ATGGGCGGAATGGTCTCTTTC (forward) and TGGGGACCTTGTCTTCATCAT (reverse), *Fasn*; GGAGGTGGTGATAGCCGGTAT (forward) and TGGGTAATCCATAGAGCCCAG (reverse), *Sqle*; ATAAGAAATGCGGGGATGTCAC (forward) and ATATCCGAGAAGGCAGCGAAC (reverse).

#### Flow cytometry

To assess live/dead cell staining, cells were stained with Live/Dead Fixable Aqua Dead Cell Stain Kit (Invitrogen, L34957) or near-IR (Invitrogen, L34975) for 20 min at room temperature. Surface staining was performed at 4°C for 15 min. To assess intracellular staining, cells were fixed and permeabilized for 30 min minimum at room temperature using the Foxp3/Transcription Factor Staining Buffer Set (eBioscience, 00552300). If cytokine expression was assessed, cells were stimulated with PMA (Sigma, P1585) and ionomycin (Sigma, 19657) in the presence of GolgiPlug (BD, 555029) prior to fixation and permeabilization unless otherwise noted. Stained cells were resuspended in 0.5% PFA and kept at 4°C until acquisition. The following antibodies were used: anti-CD3 (17A2), anti-CD4 (RM4-5), anti-CD8a (53–6.7), anti-CD25 (PC61), anti-CD45.1 (A20), anti-CD45.2 (104), anti-CD62L (MEL-14), anti-PD1 (29F.1A12), anti-IFNγ (XMG1.2), anti-IL17a (TC11-18H10.1), anti-TNFα (MP6-XT22), anti-Tbet (4B10), anti-Ki67 (16A8), anti-Rorγt (Q31-378), anti-GMCSF (MP1-22E9), anti-pSTAT5 (A17016B.Rec), anti-CD71 (RI7217), and anti-CD357/GITR (DTA-1) were purchased from Biolegend. Anti-CD44 (IM7), anti-CD45 (30-F11), anti-CD69 (H1.2F3) were purchased from BD Bioscience. Anti-Foxp3 (FJK-16s) and anti-CD137/4-1BB (17B5) were purchased from eBioscience. Cell counts were determined by the addition of cell counting beads (Spherotech, PP-50-10). Flow cytometry data was acquired using a BD Celesta (BD Biosciences). Data were analyzed using FlowJo (Tree Star) software.

#### Assessing pTreg generation induction in a T cell transfer model

CD4 T cells from WT (GluA1/2/*3 fL/fL Cre* negative control) and T^ΔAMPAR^ (GluA1/2/*3 fL/fL Cre* positive) were isolated and enriched from pooled lymph nodes and spleens by negative selection total CD4 magnetic bead kit (Miltenyi Biotech, 130-104-454). Naive CD4 cells were then flow sorted by CD4^+^, CD45RB^hi^, CD62L^+^, CD44^−^, CD25^−^. 4 × 10^5^ naive T cells were injected intraperitoneally (*i.p*.) into lymphopenic Rag2^−/−^ mice. pTreg generation was assessed by the isolation and intracellular Foxp3 staining of mesenteric lymph node, peripheral lymph node, and spleen cells at week 3 post-transfer.

#### *In vitro* iTreg suppression assay

Naive CD4 cells were isolated from WT (GluA1/2/*3 fL/fL* Cre negative control) and T^ΔAMPAR^ (GluA1/2/3 *fL/fL* Cre positive) lymph nodes and spleens using magnetic bead-based purification according to the manufacturer’s instructions (Miltenyi Biotech, 130-104-453). Cells were plated in iTreg differentiation conditions as described above for 3 days. For the suppression assay, C57Bl/6 CD8 T cells were isolated from lymph nodes and spleens using a magnetic bead-based purification kit according to the manufacturer’s instructions (Miltenyi Biotech, 130-117-044). Isolated CD8 T cells were then incubated in 5uM CellTrace violet dye (Invitrogen, C34571) for 20 min at 37°C. CTV-labeled CD8 T cells were washed twice in 1X PBS (Quality Biological, 114-058-101) supplemented with 1% FBS and 2 mM EDTA to remove excess dye. Dynabead mouse-T activator CD3/28 beads (Gibco, 11456D) were added to the CTV-labeled CD8 T cell suspension at a final ratio of 2 beads:1 cell. Unlabeled CD4 cells under iTreg differentiation culture were harvested, counted, and co-cultured with CTV-labeled CD8 T cells at ratios of 2:1, 1:1, 1:2, 1:4, 1:8, 1:16 iTreg:CD8 T cell in a round bottom 96 well plate. Unstimulated CTV-CD8 T cells were plated as a negative control and stimulated CTV-CD8 T cells were plated as a positive control. Proliferation of CTV-labeled CD8 T cells was determined by flow cytometry 3 days after co-culture.

#### *In vivo* suppression assay

nTregs were isolated and enriched from lymph nodes and spleens from WT and T^ΔAMPAR^ mice using a magnetic bead-based purification according to the manufacturer’s instructions (Miltenyi Biotech, 130-091-041). Naive CD4 cells were isolated from B6 WT 45.1 lymph nodes and spleens using magnetic bead-based purification according to the manufacturer’s instructions (Miltenyi Biotech, 130-104-453). Cells were then mixed at a 4:1 naive CD4 to nTreg ratio for WT and T^ΔAMPAR^ groups and adoptively transferred retroorbitally (*r.o.*) into Rag2^−/−^ host mice. Host mice were sacrificed 7 days after transfer and spleens were isolated and quantified as described.^33^ Splenocytes were further subjected to flow cytometry staining and analysis.

#### Bulk RNAseq and data analysis

WT (GluA1/2/*3 fL/fL Cre* negative control) and T^ΔAMPAR^ (GluA1/2/*3 fL/fL Cre* positive) naive CD4 T cells were activated and cultured under iTreg polarizing conditions for 3 days as previously described. WT and T^ΔAMPAR^ T cells were counted by trypan blue and frozen at ∼1 × 10^6^ cells/sample in 300 μL RNAprotect buffer (QIAGEN, 76526). Samples were pooled and duplicate samples per group were sequenced. RNA-sequencing analysis was performed by Admera Health (South Plainfield, NJ). Next Gen sequencing libraries were prepared using the NEBNext Ultra II Non-Directional RNA Library Prep Kit for Illumina (New England BioLabs Inc., Massachusetts, USA) according to the manufacturer’s recommendations. Final libraries quantity was assessed by Qubit 2.0 (ThermoFisher, Massachusetts, USA) and quality was assessed by TapeStation HSD1000 ScreenTape (Agilent Technologies Inc., California, USA). Final library size was about 450 bp with an insert size of about 300 bp. Illumina 8-nt dual-indices were used. Equimolar pooling of libraries was performed based on QC values and sequenced on an Illumina: Novaseq S4 (Illumina, California, USA) with a read length configuration of 150 PE for 40M PE reads per sample (20M in each direction). FastQC v0.11.8 was used to check the quality of raw reads. Trimmomatic (version 0.38) was used to cut adaptors and remove low quality bases. The clean reads were then mapped onto hg38 using STAR Aligner version 2.7.1a. FeatureCounts version 1.6.0 was used to count the number of mapped reads in genes. Picard (version 2.20.4) (https://broadinstitute.github.io/picard/) was used to collect metrics on the distribution of the bases within the transcripts in order to check mapping quality. StringTie version 2.0.4 was used to calculate FPKM (Fragments Per Kilobase per Million reads mapped) values. R package DESeq2 version 1.26.0 was used to find out the differentially expressed genes (DEG). GSEA was performed using public gene sets (HALLMARK, KEGG, and GO).^68^

#### Western blot

Pellets were lysed in RIPA buffer (Sigma, RO278) supplemented with a protease inhibitor cocktail (Thermo Scientific, 78440. Protein quantification was determined by a Pierce BCA assay (Thermo Scientific, 23225) and normalized across samples. Samples were run on 4–15% Mini-Protean TGX SDS-PAGE gels (Biorad, 4561085) and transferred to methanol-activated PVDF membranes (Biorad, 1620177). Immunoblot was performed using a standard protocol. Probes included: anti-pSMAD3 (Cell signaling, 9520), anti-total SMAD2/3 (Cell signaling, 3102), anti-pmTOR (Cell signaling, 2971), anti-total mTOR (Cell signaling, 2983), anti-Srebp1 (Abcam, ab3259), anti-Srebp2 (Abcam, ab30682), anti-β-tubulin (Cell signaling, 86298) and anti-β-actin (Cell signaling, 4970). Secondary antibodies were HRP-conjugated anti-mouse (Cell signaling, 7076) and anti-rabbit (Cell signaling, 7074). SuperSignal ECL west femto was used for development of blots (Thermo Scientific, 34095). Densitometry analysis was performed using ImageJ.^61^

#### Metabolic studies

For fatty acid and cholesterol content, cells were stained for flow cytometry with BODIPY 493/503 (Thermo Fisher, D3922) and Filipin III (Sigma, SAE0087), respectively. For the metabolomic study, *in vitro* differentiated WT and T^ΔAMPAR^ iTreg cells were submitted to Metware Biotechnology for widely targeted metabolomics. Real-time measurements of extracellular acidification rate (ECAR) and oxygen consumption rate (OCR) were performed using an XFe-96 Bioanalyzer (Agilent Technologies). T cells (2 × 10^5^ cells per well; minimum of four wells per sample) were spun into previously poly-*d*-lysine-coated 96-well plates (Seahorse) in complete RPMI-1640 medium. ECAR was measured in RPMI medium in basal condition and in response to 25 mM glucose, 1 μM oligomycin, and 50 mM of 2-DG (all from Sigma Aldrich). OCR was measured in RPMI medium supplemented with 25 mM glucose, 2 mM L-glutamine, and 1 mM sodium pyruvate, under basal condition and in response to 1 μM oligomycin, 1.5 μM of carbonylcyanide-4-(trifluoromethoxy)-phenylhydrazone (FCCP) and 1 μM of rotenone and antimycin (all from Sigma Aldrich).

### Quantification and statistical analysis

All numerical data were processed using Graph Pad Prism 7. Data are expressed as the mean ± the SEM, or as stated. Statistical comparisons were made using an unpaired Student’s t test or ANOVA with Šídák’s multiple comparison tests where 0.05 was considered significant and a normal distribution was assumed. The *p* values are represented as follows: ∗*p* < 0.05; ∗∗*p* < 0.01; ∗∗∗*p* < 0.001, ∗∗∗∗*p* < 0.0001.
