# Supplementary material for: The ionotropic AMPA receptor contributes to autoimmunity via altered regulatory T cell differentiation
Source: iScience. 2025 Nov 27;29(1):114267. doi: 10.1016/j.isci.2025.114267 (PMC12757582; doi:10.1016/j.isci.2025.114267)
Supplement: Document S1. Figures S1–S3 [file mmc1.pdf]

iScience, Volume *29*

## Supplemental information

# The ionotropic AMPA receptor contributes to autoimmunity via altered regulatory T cell differentiation

**Marisa Mitchell-Flack, Makenzie Higgins, Ying Zheng, Ada Tam, Hana Goldschmidt, Hiroshi Nishio, Bian Liu, Christopher M. Cherry, Michael Patatanian, Richard Blosser, Sung Soo Mun, Aditya Suru, Richard Huganir, Hong Yu, and Drew Pardoll**

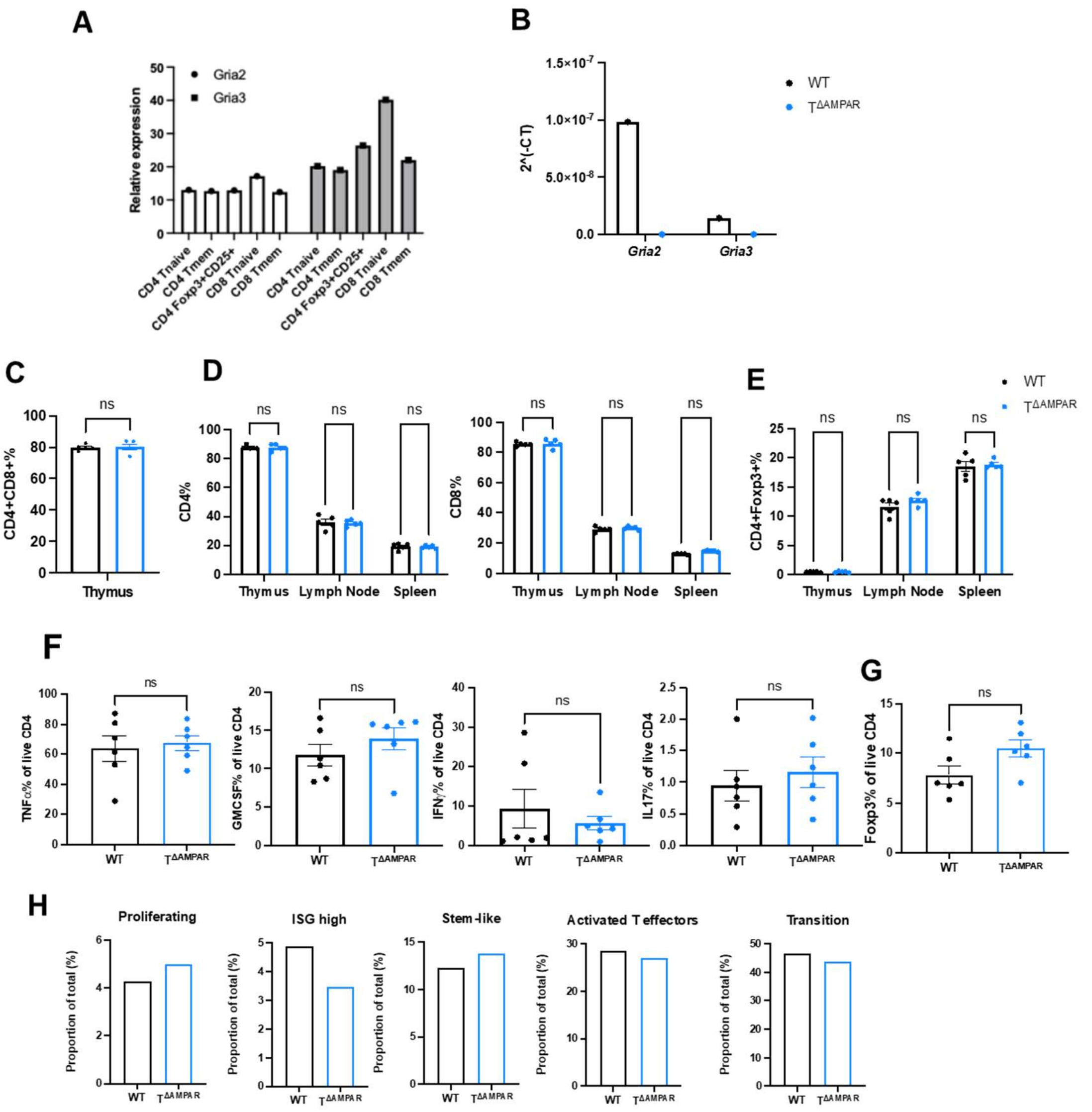


**Figure S1:** $T^{\Delta AMPAR}$ mice develop normally and have normal Th subsets within lymph nodes during EAE (related to Figure 1).

(**A-B**) Gria2 and Gira3 expression. (**A**) Gria2 and Gria3 mRNA expression in different T cell populations in mice. Data from Immunological Genome Project (ImmGen). (**B**) Gria2 and Gria3 mRNA expression relative to β-Actin in WT and $T^{\Delta AMPAR}$ CD4 cultured under iTreg condition quantified by qPCR (mean, representative of 2 independent experiments).

(**C-E)** T cell frequencies at homeostasis among thymus, lymph node, and spleen in WT and $T^{\Delta AMPAR}$ mice quantified by flow cytometry. (**C**) Quantification of CD4+CD8+ double positive frequency in thymus. (**D**) Quantification of CD4+ and CD8+ frequency, and (**E**) CD4+Foxp3+ frequency in thymus, lymph node, and spleen (mean +/- SEM, ANOVA, representative of 2 independent experiments with 3-7 biological replicates per group).

(**F-G**) Draining lymph nodes (inguinal) from the CFA-MOG injection site were isolated on day 15. Quantification of frequency of CD4+ (**F**) TNFα, GMCSF, IFNγ, IL17, and (**G**) Foxp3 (mean +/- SEM, ANOVA, representative of more than 3 independent experiments with 4-6 biological replicates per group).

(**H)** Proportions of WT and $T^{\Delta AMPAR}$ CD4 T cells identified within scRNAseq clusters depicted in Fig. 1F. Statistical significance is represented as ns > 0.05.

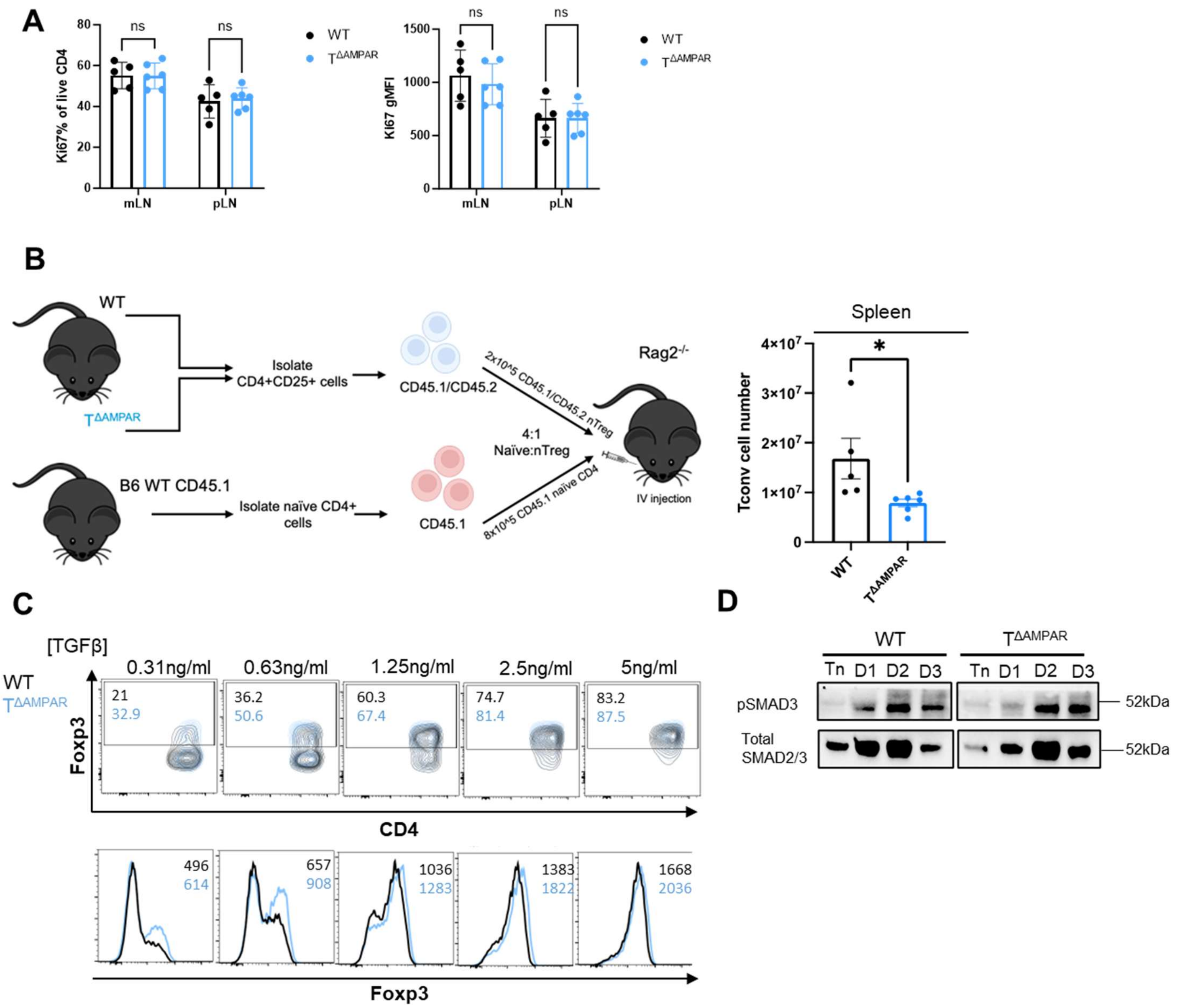


**Figure S2**: T$^{\Delta AMPAR}$ Treg differentiation, function, and signaling (related to Figure 2 and 3)

(**A**) Naïve CD4 T cells were isolated from WT and T$^{\Delta AMPAR}$ mice and adoptively transferred to Rag2-/- mice. Representative quantification of Ki67 frequency of live CD4 (left) and gMFI (right) from week 3 harvested mLN and pLN (mean +/- SEM, ANOVA, representative of 3 independent experiments).

(**B**) nTregs were isolated from WT and T$^{\Delta AMPAR}$ CD45.1/CD45.2 mice and naïve CD4 T cells were isolated from WT CD45.1 mice. For each group, cells were combined at a ratio of 4:1 naïve CD4:nTreg and co-transferred into Rag2-/- mice. Cells were recovered from the spleens of Rag2-/- mice on day 7. Representative experimental protocol (left) and quantification (right) of cell number of WT CD45.1 CD4 cells is depicted (mean +/- SEM, ANOVA, representative of 3 independent experiments).

(**C**) WT and T$^{\Delta AMPAR}$ naive CD4 T cells were cultured under iTreg conditions with a titration of TGFβ Representative flow plots (top) and histograms (bottom) of Foxp3 expression depicted from low to high concentrations of TGFβ (left to right).

(**D**) Immunoblot for phosphorylated SMAD3 (pSMAD3) in WT and T$^{\Delta AMPAR}$ CD4+ naïve T cells and cells collected on day 1, 2, and 3 of in vitro iTreg differentiation (representative of two independent experiments).

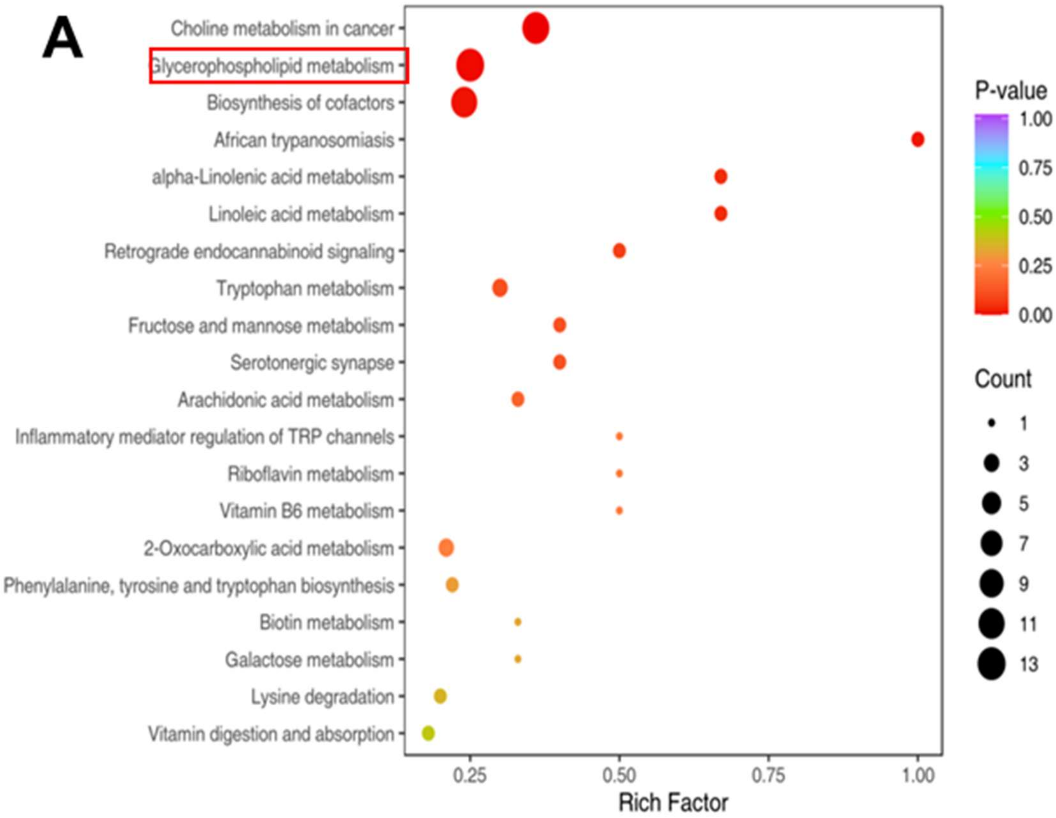


**Figure S3**: Enhanced $T^{\Delta AMPAR}$ iTreg differentiation and function is associated with upregulation of lipid metabolism (related to Figure 3)

(**A**) KEGG pathway enrichment diagram of differential metabolites generated from untargeted metabolomic detection of WT and $T^{\Delta AMPAR}$ CD4 cells under iTreg culturing conditions.
